# Multidrug-resistant *Acinetobacter* outbreak in spinal cord unit identified and stemmed through infection prevention epidemiologic efforts

**DOI:** 10.1017/ash.2023.359

**Published:** 2023-09-29

**Authors:** Sean O’Neil, Delvina Ford, Erica Beck, Theresa Carroll

## Abstract

**Background:** Drug-resistant pathogens are a significant source of increased cost and patient complications in long-term and/or congregate care settings. Once introduced, depending on the environmental niche in which they establish and the mechanisms they employ for survival, they can be difficult to eradicate. We report the details of an epidemiologic investigation of a multidrug-resistant *Acinetobacter baumannii* (MDR-A) outbreak in a spinal cord intervention (SCI) unit within a Veterans Affairs facility in San Antonio, Texas, that was identified after back tracing a positive wound culture from a long-term resident. **Methods:** All MDR-A isolates were matched to the patients harboring them. Their clinical, epidemiologic, and geographic histories within our facility were traced. All potentially shared characteristics between cases were evaluated closely. **Results:** In total, 5 cases were determined to be likely connected over a period of ~18 months starting December 2020. The extant isolates underwent molecular evaluation and were genetically related. Patient activity was traced by the infection prevention team to identify potential sources of transmission. Environmental sampling after standard cleaning found a common strain on a shower trolley shared by these patients. Following focused cleaning of this and other shared locations, no new related isolates have been identified from patient or environmental samples. **Conclusions:** In this case, investigation by the infection prevention team of a single multidrug-resistant organism led to identification and eradication of a potential pathogen. Despite standard cleaning processes, a likely shared fomite was identified and decontaminated, thereby preventing future infections. This case exemplifies the value of thorough epidemiologic study paired with modern molecular methods of identification.

**Figure:**
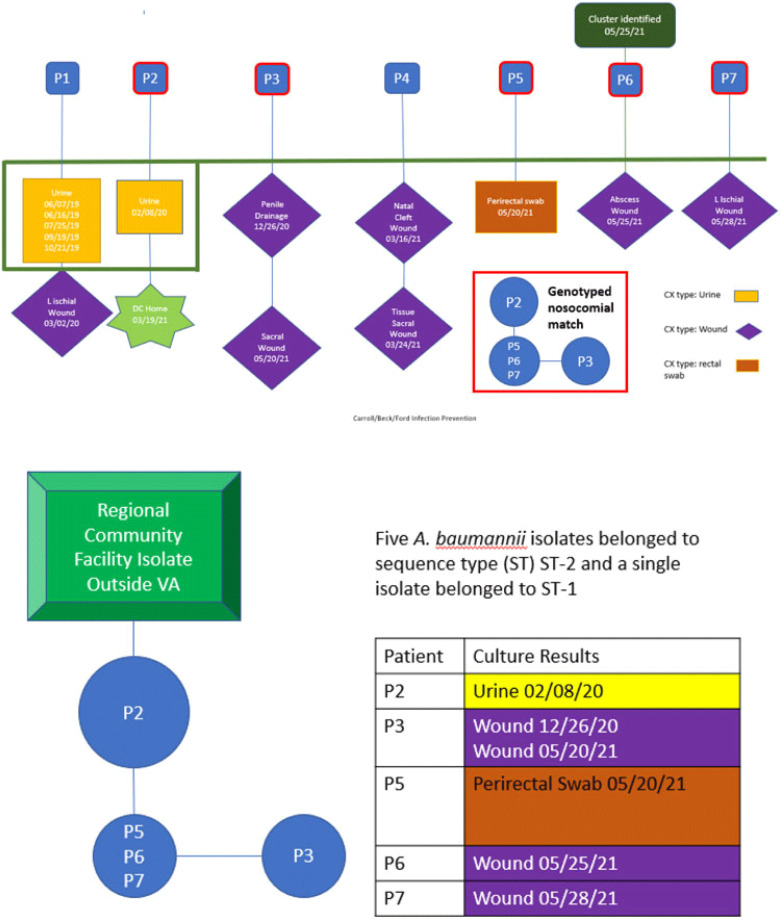

**Disclosures:** None

